# MAZ51 Blocks the Tumor Growth of Prostate Cancer by Inhibiting Vascular Endothelial Growth Factor Receptor 3

**DOI:** 10.3389/fphar.2021.667474

**Published:** 2021-04-20

**Authors:** Aya Yamamura, Md Junayed Nayeem, Hiroyuki Muramatsu, Kogenta Nakamura, Motohiko Sato

**Affiliations:** ^1^ Department of Physiology, Aichi Medical University, Nagakute, Japan; ^2^ Department of Urology, Aichi Medical University, Nagakute, Japan

**Keywords:** MAZ51, PC-3, prostate cancer, vascular endothelial growth factor, VEGF-C, VEGFR-3

## Abstract

Vascular endothelial growth factor (VEGF) signaling plays a critical role in the carcinogenesis and tumor development of several cancer types. However, its pathological significance in prostate cancer, one of the most frequent and lethal malignancies in men, remains unclear. In the present study, we focused on a pathological role of the VEGF receptors (VEGFRs), and examined their expression and effects of MAZ51 (an inhibitor of the tyrosine kinase of VEGFR-3) on cell proliferation, migration, and tumor growth in human prostate cancer cells. The expression level of VEGFR-3 was higher in androgen-independent and highly metastatic prostate cancer PC-3 cells than in other prostate PrEC, LNCaP, and DU145 cells. In PC-3 cells, VEGFR-3 and Akt were phosphorylated following a stimulation with 50 ng/ml VEGF-C, and these phosphorylations were blocked by 3 μM MAZ51. Interestingly, PC-3 cells themselves secreted VEGF-C, which was markedly larger amount compared with PrEC, LNCaP, and DU145 cells. MAZ51 reduced the expression of VEGFR-3 but not VEGFR-1 and VEGFR-2. The proliferation of PC-3 cells was inhibited by MAZ51 (IC_50_ = 2.7 μM) and VEGFR-3 siRNA, and partly decreased by 100 nM GSK690693 (an Akt inhibitor) and 300 nM VEGFR2 Kinase Inhibitor I. MAZ51 and VEGFR-3 siRNA also attenuated the VEGF-C-induced migration of PC-3 cells. Moreover, MAZ51 blocked the tumor growth of PC-3 cells in a xenograft mouse model. These results suggest that VEGFR-3 signaling contributes to the cell proliferation, migration, and tumor growth of androgen-independent/highly metastatic prostate cancer. Therefore, the inhibition of VEGFR-3 has potential as a novel therapeutic target for the treatment for prostate cancer.

## Introduction

The number of patients diagnosed with prostate cancer is increasing yearly. Recent cancer statistics in the United States revealed an estimated 191,930 new cases of prostate cancer and 33,330 deaths (Siegel et al., 2020). The 5-year survival rate of regional prostate cancer is more than 99%, whereas that of the metastatic stage is only 31% (Siegel et al., 2020). Prostate cancer is generally diagnosed by an elevated prostate-specific antigen (PSA) level in the blood and a histopathological examination of prostate biopsy specimens. Malignancy grades are evaluated according to the Gleason score (Sathianathen et al., 2018). In the early stages of prostate cancer, tumor growth is dependent on androgens. Therefore, androgen deprivation therapy is an effective strategy for achieving tumor regression. However, long-term treatment results in castration-resistant prostate cancer, which is less sensitive to androgens. Tumor progression in the metastatic stage is also independent of androgens (Hughes et al., 2005; Flourakis and Prevarskaya, 2009). The treatment of androgen-independent prostate cancer is still clinically challenges (Sathianathen et al., 2018).

The vascular endothelial growth factor (VEGF) family and its receptors (VEGFRs) play pivotal roles in the regulation of vasculogenesis, vascular permeabilization, angiogenesis, and lymphangiogenesis during organ development (Roskoski, 2007; Ferrara and Adamis, 2016). The human VEGF family includes VEGF-A, VEGF-B, VEGF-C, VEGF-D, and placental growth factor. VEGFRs consist of three receptor tyrosine kinases: VEGFR-1, VEGFR-2, and VEGFR-3. Each VEGF binds to its specific VEGFR, which facilitates their dimerization and phosphorylation. VEGF signals mainly activate downstream cascades, such as the mitogen-activated protein kinase [MAPK; e.g., extracellular signal-regulated kinase 1/2 (ERK1/2) and p38] and Akt pathways (Ferrara and Adamis, 2016). Previous studies reported that the constitutive knockout of VEGFRs showed embryonic lethality on embryonic days 8.5–9.5 due to endothelial dysfunction and cardiovascular defects (Simons et al., 2016). VEGFRs are predominantly expressed in vascular and lymphatic endothelial cells, but have also been detected in other types of cells (Simons et al., 2016).

VEGFs and VEGFRs have been implicated in a number of diseases including cancer, rheumatoid arthritis, atherosclerosis (Shibuya and Claesson-Welsh, 2006; Ferrara and Adamis, 2016), lung diseases (pulmonary hypertension and pulmonary fibrosis) (Al-Husseini et al., 2015; Barratt et al., 2018), and hematological disorders (myeloproliferative neoplasms, myelodysplastic syndromes, and acute myeloid leukemia) (Yang et al., 2018). VEGFs and VEGFRs play important roles in angiogenesis related to the pathogenesis of solid tumors. Furthermore, enhanced VEGF signaling has been shown to mediate the metastasis and progression of several cancer types (Ferrara and Adamis, 2016; Roskoski, 2017). Although VEGFs and VEGFRs were previously shown to be expressed in prostate cancer (Su et al., 2007; Roberts et al., 2013), their pathological roles have not yet been elucidated.

In the present study, the expression of VEGFRs was compared among human prostate epithelial PrEC cells, androgen-dependent/weakly metastatic prostate cancer LNCaP cells, androgen-independent/highly bone metastatic prostate cancer PC-3, and androgen-independent/moderate brain metastatic prostate cancer DU145 cells. MAZ51 is a tyrosine kinase inhibitor of VEGFR-2 and VEGFR-3, and preferentially inhibits the phosphorylation of VEGFR-3 (lower concentrations at ∼5 μM) over that of VEGFR-2 (higher concentrations at ∼50 μM) (Kirkin et al., 2001). The pharmacological effects of lower concentrations of MAZ51 (∼3 μM; an inhibitor of the tyrosine kinase of VEGFR-3) on cell proliferation, migration, and tumor growth in PC-3 cells and a xenograft carcinoma transplantation mouse model were also examined. The results obtained clearly demonstrated that VEGFR-3 was specifically up-regulated in PC-3 cells and thereby MAZ51 blocked the tumor growth of prostate cancer *via* the inhibition of proliferation and migration of these cells.

## Materials and Methods

### Cell Culture

The human prostate epithelial cell line, PrEC, was purchased from Lonza (Walkersville, MD, United States). PrEC cells (passages 5–11) were cultured in PrEGM BulletKit medium (Lonza) supplemented with 10% fetal bovine serum (FBS; GIBCO/Invitrogen, Grand Island, NY, United States) and 100 U/ml penicillin plus 100 μg/ml streptomycin (GIBCO/Invitrogen) at 37°C. The human prostate cancer cell lines, LNCaP and DU145 (originally from the American Type Culture Collection, Manassas, VA, United States), were provided by Dr Hirotsugu Uemura (Kindai University, Osaka, Japan) (Muramatsu et al., 2019). The human prostate cancer cell line, PC-3, was obtained from the Cell Resource Center for Biomedical Research, the Institute of Development, Aging and Cancer, Tohoku University (Sendai, Japan). LNCaP, PC-3, and DU145 cells (passages 5–11) were cultured in RPMI-1640 medium (Sigma-Aldrich, St. Louis, MO, United States) supplemented with 10% FBS and 100 U/ml penicillin plus 100 μg/ml streptomycin at 37°C. Experiments using VEGF-C stimulation were performed after the exposure to RPMI-1640 medium containing 1% FBS for 4 h to prevent enhanced phosphorylation and starvation stress.

### Western Blotting

The protein fraction was extracted from the homogenates of prostate cells using RIPA buffer (Pierce Biotechnology, Rockford, IL, United States), as described previously (Yamamura et al., 2019a; Yamamura et al., 2019b). In brief, the extracted protein (20 μg/lane) was added to an 8% acrylamide gel and transferred to an Immobilon-P PVDF membrane (Millipore, Bedford, MA, United States). The membrane was blocked with Tris-buffered saline containing 5% bovine serum albumin (Sigma-Aldrich) and then incubated with the primary antibody (1:1000) for VEGFR-1 (#2893, Cell Signaling Technology, Danvers, MA, United States), *p*-VEGFR-1 (Tyr1333, SAB4504006, Sigma-Aldrich), VEGFR-2 (#2479, Cell Signaling Technology), *p*-VEGFR-2 (Tyr996, #2474, Cell Signaling Technology), VEGFR-3 (#79-633, ProSci, Poway, CA, United States; sc-28297 and sc-321, Santa Cruz Biotechnology, Santa Cruz, TX, United States), *p*-Akt (Ser473, #9271, Cell Signaling Technology), Akt (#9272, Cell Signaling Technology), *p*-ERK1/2 (Thr202/Tyr204, #9101, Cell Signaling Technology), ERK1/2 (#9102, Cell Signaling Technology), *p*-p38 (Thr180/Tyr182, #9211, Cell Signaling Technology), or p38 (#9211, Cell Signaling Technology). Immunoblotted membranes were treated with an anti-rabbit or anti-mouse horseradish peroxidase-conjugated IgG secondary antibody (1:5000; #1706515 or #1706516, BioRad, Hercules, CA, United States). Blotting signals were detected using the ImmunoStar LD (Wako Pure Chemicals, Osaka, Japan). These images were analyzed using the Amersham Imager 600 system (GE Healthcare Life Sciences, Pittsburgh, PA, United States). Protein expression was normalized using a β-actin antibody (1:5000; AC-74, Sigma-Aldrich).

### Transfection of siRNA

PC-3 cells (1 × 10^5^ cells/well) were transiently transfected with the siRNA (50 and 100 nM) of the VEGFR-3 (s5296, Silencer Select, Ambion/Applied Biosystems, Auatin, TX, United States) or negative control (Silencer Negative Control #1, Ambion/Applied Biosystems) using Lipofectamine RNAiMax transfection reagent (Invitrogen), as described previously (Yamamura et al., 2019b). siRNA experiments were performed 48 h after transfection.

### Co-Immunoprecipitation Assay

Co-immunoprecipitation was performed with a modification using a Co-Immunoprecipitation kit (Pierce Biotechnology) (Sakima et al., 2018). PC-3 cells were lysed in immunoprecipitation lysis/wash buffer with a protease inhibitor mixture (Sigma-Aldrich). Homogenates were centrifuged (15,000 × g, 4°C, 25 min), and the supernatant was precleared with control resin (4°C, 1 h). Precleared lysates (∼100 μg of protein) were incubated with AminoLink Plus Coupling Resin, with which the VEGFR-3 antibody was immobilized at 4°C for 12 h. Incubated lysates were finally subjected to 7.5% sodium dodecyl sulfate-polyacrylamide gel electrophoresis (SDS-PAGE). The blots were incubated with a *p*-Tyr (PY20) antibody (1:1000; sc-508, Santa Cruz Biotechnology) at 4°C for 12 h, and then treated with the anti-rabbit horseradish peroxidase-conjugated IgG antibody (1:2000) at 4°C for 1 h. Chemiluminescence was detected using a similar method to that for Western blotting.

### ELISA Assay

The amount of VEGF-C secreted from prostate cells was quantitatively measured using a human VEGF-C Quantikine ELISA kit (R&D Systems, Minneapolis, MN, United States), according to the manufacturer’s instructions.

### Cell Viability Assay

Prostate cells (3 × 10^4^ cells/well) were subcultured in 96-well plates and incubated at 37°C (Yamamura et al., 2019b). These cells were exposed to culture medium including vehicle (0.1% dimethyl sulfoxide, DMSO) or the drug for 48 h. Cell viability was evaluated using Cell Counting Kit-8 (Dojindo Laboratories, Kumamoto, Japan) based on the 3-(4,5-dimethyl-2-thiazolyl)-2,5-diphenyl-2*H*-tetrazolium bromide (MTT) assay. The results obtained were quantified colorimetrically as absorbance at 450 nm using a SpectraMax M3 (Molecular Devices, San Jose, CA, United States).

### Cell Proliferation Assay

Prostate cells (3 × 10^4^ cells/well) were subcultured in 96-well plates and incubated at 37°C (Yamamura et al., 2019b). Drug application was performed in the same manner as that described for the MTT assay. The proliferation of PC-3 cells was evaluated using the Cell Proliferation ELISA, BrdU (colorimetric) kit (Roche Diagnostics, Mannheim, Germany) based on the bromodeoxyuridine (BrdU) incorporation assay. Colorimetric quantification as absorbance at 370 nm was measured using a similar method to that for the MTT assay.

### Cell Migration Assay

PC-3 cells (1 × 10^5^ cells/well) were seeded on a six-well Transwell plate with a membrane pore size of 8 μm (#3428, Corning, Tewksbury, MA, United States), as described previously (Yamamura et al., 2019b). Percentage of FBS in culture medium filled in the upper and lower chambers was reduced to 1% to prevent cell proliferation. These cells were then exposed to culture medium including vehicle (0.1% DMSO) or the drug in the lower chamber for 18 h. Transwell inserts were fixed in 4% paraformaldehyde and stained with 1% crystal violet. The number of migrated cells was counted from digital microscopic images of the Transwell inserts.

### Xenograft Mouse Model

All experiments were approved by the Ethics Committee of Aichi Medical University (2019-15), and conducted in accordance with the Guide for the Care and Use of Laboratory Animals of the Japanese Pharmacological Society. PC-3 cells were suspended in 1.0 ml physiological saline with matrigel. PC-3 cells (2.0×10^7^ cells/0.1 ml saline) were then injected into the subcutaneous tissue of male BALB/c-*nu/nu* mice (5 weeks, Japan SLC, Hamamatsu, Japan). Drugs were suspended in 0.5 ml physiological saline and applied subcutaneously around the tumors every days. Tumor volume [defined as (length×width^2^)/2] and weight were measured every week.

### Drugs

Pharmacological reagents were obtained from Sigma-Aldrich, except for VEGF-C (Wako Pure Chemicals), GSK690693 {4-[2-(4-amino-1,2,5-oxadiazol-3-yl)-1-ethyl-7-{[(3S)-3-piperidinylmethyl]oxy}-1H-imidazo [4,5-c]pyridin-4-yl]-2-methyl-3-butyn-2-ol} (Selleck Biotech, Tokyo, Japan), and VEGFR2 Kinase Inhibitor I ((Z)-3-{[2,4-dimethyl-3-(ethoxycarbonyl)pyrrol-5-yl]methylidenyl}indolin-2-one) (Calbiochem, La Jolla, CA, United States). VEGF-C was dissolved in sterile water at a concentration of 10 μg/ml as a stock solution. MAZ51 [3-(4-dimethylaminonaphthalen-1-ylmethylene)-1,3-dihydroindol-2-one] and VEGFR2 Kinase Inhibitor I were dissolved in DMSO at a concentration of 10 mM as a stock solution.

### Statistical Analysis and Miscellaneous Procedures

Pooled data are shown as the mean ± S.D. The sample size was determined in advance. The significance of differences between two groups was assessed by the Student’s *t*-test using the BellCurve for Excel software (version 3.20; Social Survey Research Information, Tokyo, Japan). The significance of differences among groups was evaluated by Scheffé’s test following a one-way analysis of variance (ANOVA) or the non-parametric Mann-Whitney U-test using the same software. A *p* value of <0.05 was considered to be significant. Other miscellaneous procedures are previously described (Sato et al., 2011).

## Results

### Expression of VEGF Receptors in Human Prostate Cancer Cells

**FIGURE 1:**
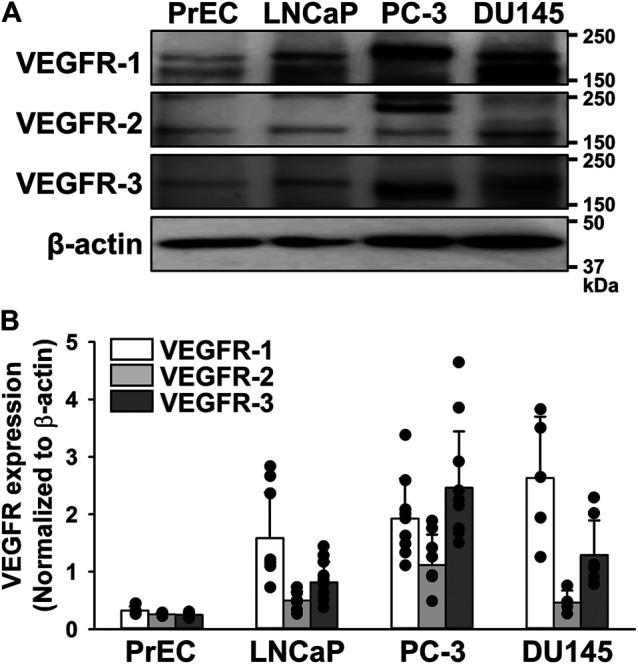
Up-regulation of VEGFR-3 in human prostate cancer PC-3 cells. The protein expression of VEGFRs was examined in human prostate epithelial PrEC cells and prostate cancer LNCaP (androgen-dependent/weakly metastatic), PC-3 (androgen-independent/highly bone metastatic), and DU145 (androgen-independent/moderate brain metastatic) cells by Western blotting. **(A)** Representative blots of VEGFR-1, VEGFR-2, and VEGFR-3 in PrEC, LNCaP, PC-3, and DU145 cells. β-actin was used as an endogenous marker. **(B)** Expression levels of VEGFR-1, VEGFR-2, and VEGFR-3 in PrEC, LNCaP, PC-3, and DU145 cells (*n* = 4–11). The expression levels of VEGFRs were normalized by that of β-actin. Note that the expression level of VEGFR-3 was higher in PC-3 cells than in PrEC, LNCaP, and DU145 cells. Data are presented as means ± S.D.

The protein expression of VEGFRs (VEGFR-1, VEGFR-2, and VEGFR-3) was examined in human prostate epithelial PrEC cells and prostate cancer LNCaP (androgen-dependent/weakly metastatic), PC-3 (androgen-independent/highly bone metastatic), and DU145 (androgen-independent/moderate brain metastatic) cells by Western blotting. The expression level of VEGFR-1 was higher in LNCaP, PC-3, and DU145 cells than in PrEC cells (Figure 1). The expression levels of VEGFR-2 and VEGFR-3 were higher in PC-3 cells than in LNCaP and DU145 cells, but were very low in PrEC cells. These results indicate that the VEGFR-3 protein was more strongly expressed in PC-3 cells than in PrEC, LNCaP, and DU145 cells. The magnitude of increase was larger in VEGFR-3 (9.85-fold) than VEGFR-1 (5.95-fold) and VEGFR-2 (4.29-fold) in PC-3 cells.

**FIGURE 2:**
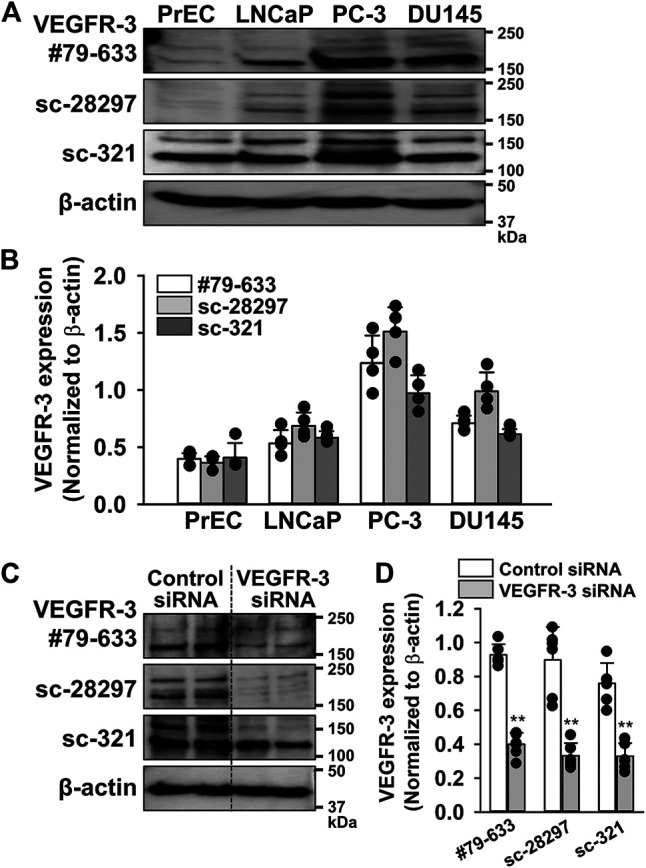
Abundant expression of VEGFR-3 in PC-3 cells. The expression of VEGFR-3 protein was examined in PrEC, LNCaP, PC-3, and DU145 cells by Western blotting using three different antibodies for VEGFR-3. **(A)** Representative blots of VEGFR-3 in PrEC, LNCaP, PC-3, and DU145 cells using three different VEGFR-3 antibodies: #79-633 (ProSci), sc-28297, and sc-321 (Santa Cruz Biotechnology). **(B)** Expression level of VEGFR-3 in PrEC, LNCaP, PC-3, and DU145 cells (*n* = 4). The expression level of VEGFR-3 were normalized by that of *β*-actin. **(C)** Representative blots of VEGFR-3 in PC-3 cells treated with 50 nM control or VEGFR-3 siRNA for 48 h. **(D)** Expression level of VEGFR-3 in PC-3 cells treated with control or VEGFR-3 siRNA (*n* = 6). Data are presented as means ± S.D. **p < 0.01 vs. control siRNA (unpaired two-tailed *t*-test).

To confirm whether the expression level of VEGFR-3 protein was higher in PC-3 cells than in other prostate cells, similar experiments were performed using three different antibodies for VEGFR-3 (#79-633, ProSci; sc-28297 and sc-321, Santa Cruz Biotechnology). These results strongly suggest that the expression of VEGFR-3 protein in PC-3 cells was the strongest among prostate cells examined (Figures 2A,B). Furthermore, the specificity of these antibodies was confirmed by the knockdown using 50 nM VEGFR-3 siRNA in PC-3 cells (Figures 2C,D). Therefore, we focused on examining a pathological role of VEGFR-3 signaling in PC-3 cells.

### MAZ51 Inhibits the VEGF-C-Induced Phosphorylation of VEGFR-3

**FIGURE 3:**
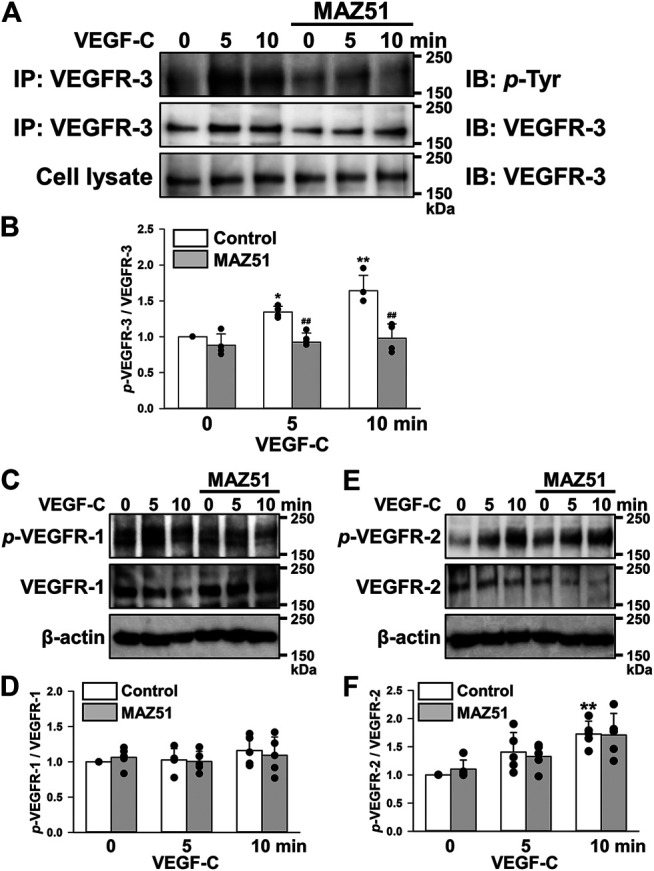
Inhibition of the VEGF-C-induced phosphorylation of VEGFR-3 by MAZ51. The phosphorylation of VEGFRs after a stimulation with VEGF-C (a ligand for VEGFR-2 and VEGFR-3) in the absence and presence of MAZ51 in human prostate cancer PC-3 cells using Western blotting and co-immunoprecipitation methods. **(A)** Representative blots of phosphorylation of VEGFR-3 stimulated by 50 ng/ml VEGF-C in the absence and presence of 3 μM MAZ51 for 4 h in PC-3 cells. **(B)** The phosphorylation level of VEGFR-3 by the VEGF-C stimulation in the absence and presence of MAZ51 in PC-3 cells (*n* = 4). **(C)** Representative blots of phosphorylation of VEGFR-1 stimulated by VEGF-C in the absence and presence of MAZ51 in PC-3 cells. **(D)** The phosphorylation level of VEGFR-1 by the VEGF-C stimulation in the absence and presence of MAZ51 in PC-3 cells (*n* = 5). **(E)** Representative blots of phosphorylation of VEGFR-2 stimulated by VEGF-C in the absence and presence of MAZ51 in PC-3 cells. **(F)** The phosphorylation level of VEGFR-2 by the VEGF-C stimulation in the absence and presence of MAZ51 in PC-3 cells (*n* = 5). Data are presented as means ± S.D. *p < 0.05, **p < 0.01 vs. 0 min; ^##^ *p* < 0.01 vs. control (unpaired two-tailed *t*-test).

The phosphorylation of VEGFRs in PC-3 cells was analyzed during a stimulation with VEGF-C (a ligand for VEGFR-2 and VEGFR-3). Analyses of VEGFR-3 phosphorylation were performed using the co-immunoprecipitation method with VEGFR-3 and *p*-Tyr antibodies. The phosphorylation of VEGFR-3 was facilitated by 50 ng/ml VEGF-C stimulation in a time-dependent manner (Figures 3A,B). The VEGF-C-induced phosphorylation of VEGFR-3 was completely blocked by the pretreatment with 3 μM MAZ51 (an inhibitor of the tyrosine kinase of VEGFR-3) for 4 h. On the other hand, VEGF-C and MAZ51 did not affect the phosphorylation of VEGFR-1 in PC-3 cells (Figures 3C,D). Although the phosphorylation of VEGFR-2 was facilitated by VEGF-C stimulation in PC-3 cells, the phosphorylation was not blocked by MAZ51 (Figures 3E,F). These results suggest that VEGFR-3 still responded to VEGF-C stimulation and its up-regulation enhanced VEGFR-3 signaling in PC-3 cells.

### Downstream Cascade of VEGFR-3 After VEGF-C Stimulation

**FIGURE 4:**
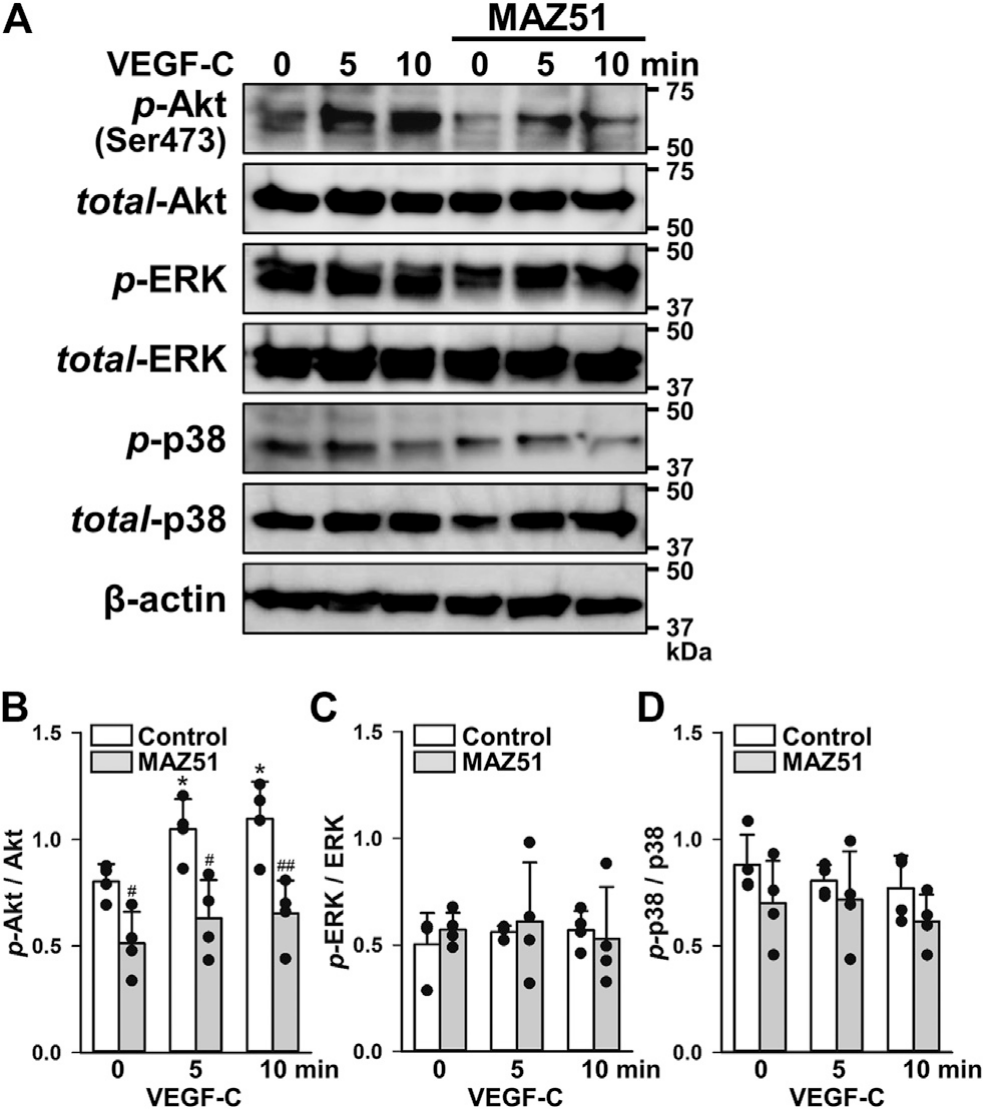
Phosphorylation pathway following VEGF-C-induced VEGFR-3 activation. The phosphorylation signaling after VEGF-C-induced VEGFR-3 activation was assayed in the absence and presence of MAZ51 in human prostate cancer PC-3 cells by Western blotting. **(A)** Representative blots of phosphorylation of Akt (Ser473), ERK1/2, and p38 after 50 ng/ml VEGF-C stimulation in the absence and presence of 3 μM MAZ51 for 4 h in PC-3 cells. **(B–D)** The phosphorylation level of Akt **(B)**, ERK1/2 **(C)**, and p38 **(D)** by the VEGF-C stimulation in the absence and presence of MAZ51 in PC-3 cells (*n* = 4). Data are presented as means ± S.D. *p < 0.05 vs. 0 min; ^#^ *p* < 0.05, ^##^ *p* < 0.01 vs. control (unpaired two-tailed *t*-test).

Since MAPK and Akt pathways are supposed to be activated by VEGFR-3 stimulation (Ferrara and Adamis, 2016), their phosphorylation levels after VEGF-C stimulation in PC-3 cells were assessed in the absence and presence of MAZ51 by Western blotting. The phosphorylation of Akt was facilitated by 50 ng/ml VEGF-C stimulation in a time-dependent manner (Figures 4A,B). The VEGF-C-induced phosphorylation of Akt was markedly blocked by the pretreatment with 3 μM MAZ51 for 4 h. On the other hand, the phosphorylation of ERK1/2 and p38 MAPKs was not affected by VEGF-C stimulation and MAZ51 (Figures 4A,C,D). These results suggest that the VEGF-C-induced VEGFR-3 activation facilitates Akt cascade rather than ERK1/2 and p38 MAPK pathways in PC-3 cells.

### Secretion of VEGF-C in Human Prostate Cancer Cells

**FIGURE 5:**
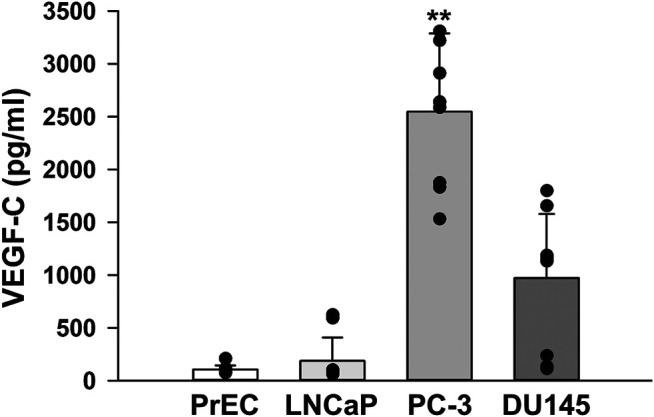
VEGF-C levels in human prostate cancer cells. The levels of VEGF-C in the culture media of human prostate epithelial PrEC cells and prostate cancer LNCaP, PC-3, and DU145 cells were measured using the human VEGF-C Quantikine ELISA kit. Note that larger amounts of VEGF-C were secreted from PC-3 cells than from PrEC, LNCaP, and DU145 cells (*n* = 10). Data are presented as means ± S.D. **p < 0.01 vs. PrEC, LNCaP, or DU145 cells (Scheffé’s test following ANOVA).

Plasma levels of VEGFs have been associated with the clinical stage, Gleason score, and serum PSA level in prostate cancer patients (Duque et al., 2006). Therefore, VEGF-C levels in the culture media of PrEC, LNCaP, PC-3, and DU145 cells were quantitatively measured using the human VEGF-C Quantikine ELISA kit. VEGF-C levels were markedly higher in the cultured medium of PC-3 cells than in those of PrEC, LNCaP, and DU145 cells (Figure 5). These results suggest that PC-3 cells secreted abundant VEGF-C, which potentially activates VEGFR-3 signaling in an autocrine manner.

### Inhibitory Effects of MAZ51 on VEGFR-3 Expression and *p*-Akt Levels

**FIGURE 6:**
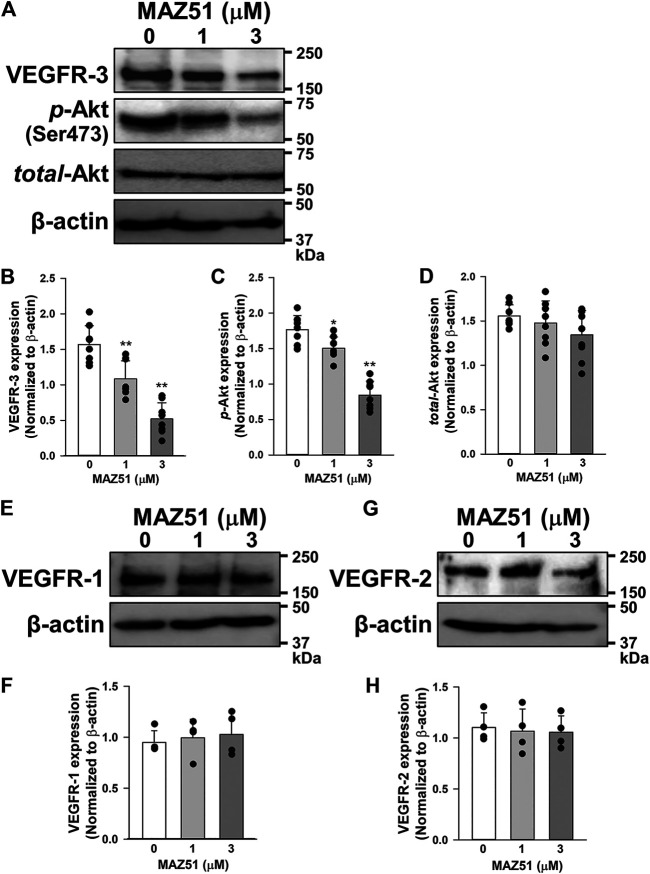
Down-regulation of VEGFR-3 expression and *p*-Akt levels by MAZ51. The effects of MAZ51 on VEGFR expression and its downstream signal pathway, Akt, were examined in human prostate cancer PC-3 cells by Western blotting. **(A)** Representative blots of VEGFR-3, *p*-Akt (Ser473), and *total*-Akt in the absence and presence of 1 and 3 μM MAZ51 for 48 h in PC-3 cells. **(B–D)** The expression levels of VEGFR-3 **(B)**, *p*-Akt **(C)**, and *total*-Akt **(D)** in the absence and presence of MAZ51 in PC-3 cells (*n* = 8). **(E,F)** Representative blots **(E)** and the expression levels **(F)** of VEGFR-1 in the absence and presence of MAZ51 in PC-3 cells (*n* = 4). **(G,H)** Representative blots **(G)** and the expression levels **(H)** of VEGFR-2 in the absence and presence of MAZ51 in PC-3 cells (*n* = 4). These expression levels were normalized by that of β-actin. Data are presented as means ± S.D. *p < 0.05, **p < 0.01 vs. 0 μM MAZ51 (Scheffé’s test following ANOVA).

The effects of long-term treatment of MAZ51 on VEGFR expression were examined in PC-3 cells by Western blotting. Interestingly, 48 h treatment of MAZ51 decreased expression of VEGFR-3 in a concentration-dependent manner (Figures 6A,B). Since Akt is a downstream signal pathway of VEGFR-3 (Ferrara and Adamis, 2016), the phosphorylation of Akt in PC-3 cells was assessed in the absence and presence of MAZ51 by Western blotting. The phosphorylation of Akt was detected in PC-3 cells even without the ligand stimulation (Figures 6A,C). It was also attenuated by the treatment with 1 and 3 μM MAZ51 for 48 h. There was no significantly difference on total expression level of Akt among all groups (Figures 6A,D). On the other hand, MAZ51 did not affect the expression of VEGFR-1 and VEGFR-2 in PC-3 cells (Figures 6E–H). These results indicate that MAZ51 inhibited the enhanced signal pathway of VEGFR-3 and Akt in PC-3 cells.

### Antiproliferative Effects of MAZ51 in Human Prostate Cancer Cells

**FIGURE 7:**
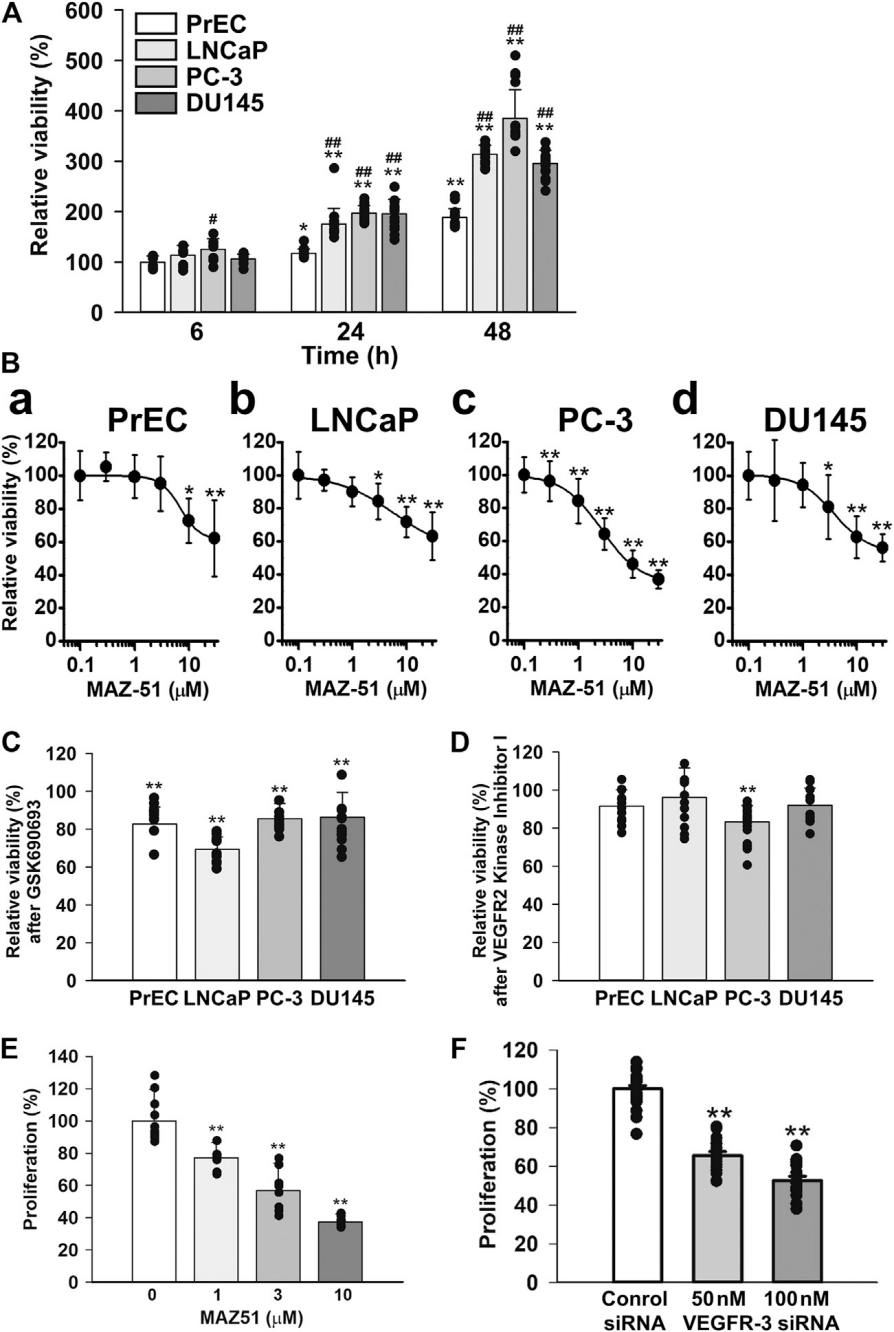
Inhibitory effects of MAZ51 on the proliferation of human prostate cancer cells. The effects of MAZ51 on the cell viability and proliferation of human prostate epithelial PrEC cells and prostate cancer LNCaP, PC-3, and DU145 cells using MTT and BrdU assays, respectively. **(A)** Time-dependent cell growth of PrEC, LNCaP, PC-3, and DU145 cells (*n* = 9–16). **(B)** The effects of MAZ51 for 48 h on the viability of PrEC, LNCaP, PC-3, and DU145 cells (*n* = 8–34). The IC_50_ values of MAZ51 on the viabilities of PrEC, LNCaP, PC-3, and DU145 cells were 7.0, 6.0, 2.7, and 3.8 μM, respectively. **(C)** The effects of 100 nM GSK690693 (an Akt inhibitor) for 48 h on the viability of PrEC, LNCaP, PC-3, and DU145 cells (*n* = 12–18). **(D)** The effects of VEGFR2 Kinase Inhibitor I for 48 h on the viability of PrEC, LNCaP, PC-3, and DU145 cells (*n* = 12–24). **(E)** The effects of MAZ51 for 48 h on the proliferation of PC-3 cells (*n* = 12). **(F)** The effects of 50 and 100 nM VEGFR-3 siRNA for 48 h on the proliferation of PC-3 cells (*n* = 16–24). Data are presented as means ± S.D. *p < 0.05, **p < 0.01 vs. 6 h, 0 μM drug, or control siRNA; ^#^ *p* < 0.05, ^##^ *p* < 0.01 vs. PrEC cells (Scheffé’s test following ANOVA).

The growth of PrEC, LNCaP, PC-3, and DU145 cells was examined using a quantitative colorimetric assay kit based on the MTT test for cellular viability. In PrEC cells, cell numbers slowly increased until 48 h after the subculture (Figure 7A). The growth of LNCaP cells was markedly higher than that of PrEC cells. Furthermore, the growth of PC-3 and DU145 cells was similar to that of LNCaP cells. Next, the effects of MAZ51, GSK690693 (an Akt inhibitor), and VEGFR2 kinase Inhibitor I on cell proliferation were examined in PrEC, LNCaP, PC-3, and DU145 cells. The growth of PrEC cells was not affected at 0.3–3 μM MAZ51 for 48 h, but was slightly inhibited at MAZ51 concentrations of 10 μM and higher (Figure 7B, a). The treatment with MAZ51 moderately inhibited the growth of LNCaP and DU145 cells in a concentration-dependent manner with an IC_50_ value of 6.0 and 3.8 μM, respectively, and a Hill coefficient of 0.84 and 1.43, respectively [Figures 7B, (b, d)]. When PC-3 cells were incubated with medium containing various concentrations of MAZ51, MAZ51 inhibited cell growth in a concentration-dependent manner with an IC_50_ value of 2.7 μM and a Hill coefficient of 1.21 (Figure 7B, c). On the other hand, 100 nM GSK690693 was inhibited the growth of all prostate cells examined (Figure 7C). The treatment with 300 nM VEGFR2 kinase Inhibitor I slightly attenuated the growth of PC-3 cells but not PrEC, LNCaP, and DU145 cells (Figure 7D).

To confirm the inhibitory effects of MAZ51 in the MTT assay, the BrdU incorporation assay based on the quantitative colorimetric method for cell proliferation was performed using PC-3 cells. The proliferation of PC-3 cells decreased in a concentration-dependent manner by the treatment with 1–10 μM MAZ51 for 48 h (Figure 7E). In addition, the cell proliferation reduced by the knockdown using 50 and 100 nM VEGFR-3 siRNA for 48 h (Figure 7F). These results suggest that the proliferation of PC-3 cells was strongly regulated by the VEGFR-3 and Akt signaling.

### Inhibitory Effects of MAZ51 on the Migration of PC-3 Cells

**FIGURE 8:**
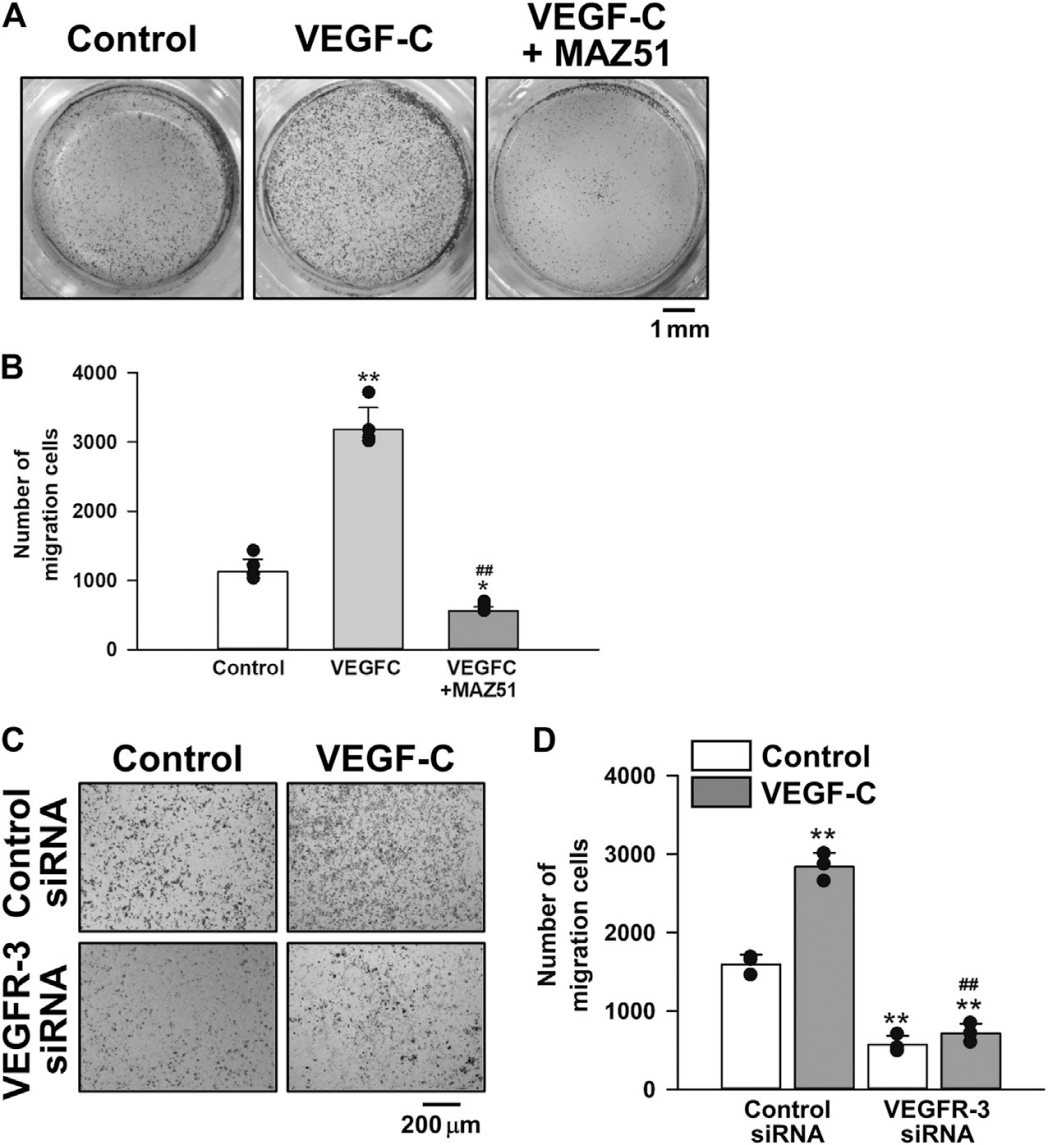
Inhibitory effects of MAZ51 and VEGFR-3 siRNA on the migration of human prostate cancer PC-3 cells. The effects of VEGF-C on the migration of human prostate cancer PC-3 cells in the absence and presence of MAZ51 or VEGFR-3 siRNA were examined using the Transwell kit. **(A)** Representative images in the absence and presence of 50 ng/ml VEGF-C and 3 μM MAZ51 for 18 h in PC-3 cells. **(B)** The effects of VEGF-C and MAZ51 on the migration of PC-3 cells (*n* = 4). **(C)** Representative images in the absence and presence of VEGF-C for 18 h in PC-3 cells treated with 50 nM VEGFR-3 siRNA for 48 h. **(D)** The effects of VEGF-C and VEGFR-3 siRNA on the migration of PC-3 cells (*n* = 3). Data are presented as means ± S.D. *p < 0.05, **p < 0.01 vs. control or control siRNA/control; ^##^ *p* < 0.01 vs. VEGF-C alone or control siRNA/VEGF-C (Scheffé’s test following ANOVA).

The effects of VEGFR-3 signaling on the migration of PC-3 cells were examined using the Transwell kit. The migration of PC-3 cells was facilitated by the stimulation with 50 ng/ml VEGF-C for 18 h. The VEGF-C-induced migration was markedly decreased in the presence of 3 μM MAZ51 (Figures 8A,B) or 50 nM VEGFR-3 siRNA (Figures 8C,D). Collectively, these *in vitro* results suggest that the VEGF-C/VEGFR-3 signal facilitated the proliferation and migration of PC-3 cells and pharmacological inhibition attenuated the pathogenic functions for growth of prostate cancer cells.

### MAZ51 Blocked the Tumor Growth of PC-3 Cells in the Xenograft Mouse Model

**FIGURE 9:**
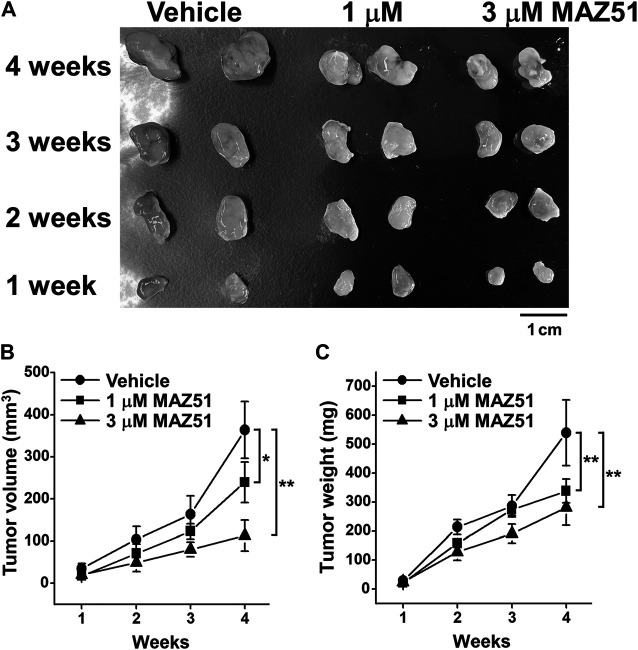
Inhibition of the tumor growth of PC-3 cells by MAZ51. The effects of MAZ51 on the tumor growth of human prostate cancer PC-3 cells were examined in a xenograft mouse model. **(A)** Representative tumors after the treatment with vehicle (0.1% DMSO), 1, or 3 μM MAZ51 for 1–4 weeks. **(B,C)** Tumor volumes **(B)** and weights **(C)** after the treatment with vehicle or MAZ51 for 1–4 weeks (*n* = 5–6). Note that the VEGFR-3 kinase inhibition by MAZ51 effectively blocked the tumor growth of prostate cancer in *ex vivo* model. Data are presented as means ± S.D. *p < 0.05, **p < 0.01 vs. vehicle control (non-parametric Mann-Whitney U-test).

To clarify the involvement of VEGFR-3 signaling in the tumor growth of human prostate cancer, the effects of MAZ51 on the tumor growth of PC-3 cells were examined using the xenograft mouse model. PC-3 cells (2 × 10^7^ cells) were subcutaneously transplanted into nude mice. Vehicle (0.1% DMSO), 1, or 3 μM MAZ51 was then subcutaneously injected around the tumors of the xenograft mouse model every day. The treatment with MAZ51 was blocked the tumor growth of PC-3 cells in a concentration-dependent manner (Figure 9A). Following the application of 3 μM MAZ51, tumor volumes were markedly lower at 2–4 weeks than at 1 week after transplantation (Figure 9B). Similarly, tumor weights were markedly lower at 2–4 weeks than at 1 week after transplantation (Figure 9C). These *ex vivo* results suggest that the VEGFR-3 kinase inhibitor effectively blocked the development of prostate cancer.

## Discussion

VEGFRs are involved in the tumor development of several cancer types, including gastrointestinal, lung, renal, thyroid, cervical, and ovarian tumors (Ferrara and Adamis, 2016; Roskoski, 2017). We also found that the expression level of a specific type of VEGFR, VEGFR-3, was higher in human prostate cancer PC-3 cells than in prostate epithelial PrEC cells and prostate cancer LNCaP and DU145 cells. Moreover, the VEGFR-3 kinase inhibitor MAZ51 or VEGFR-3 siRNA blocked the up-regulation and phosphorylation of VEGFR-3 and attenuated proliferation and migration, which inhibited the tumor growth of prostate cancer. These results strongly suggest that VEGF-C/VEGFR-3 participates in regulating cell proliferation, migration, and tumor growth in prostate cancer.

The results of the expression analyses revealed that VEGFRs were weakly expressed in human normal prostate epithelial PrEC cells. On the other hand, the expression of VEGFRs (VEGFR-1, VEGFR-2, and VEGFR-3) was up-regulated in human androgen-dependent/weakly metastatic prostate cancer LNCaP cells (4.9-, 1.9-, and 3.3-fold, respectively). Similarly, the expression of all VEGFRs was up-regulated in human androgen-independent/highly bone metastatic prostate cancer PC-3 cells (6.0-, 4.3-, and 9.8-fold, respectively) and androgen-independent/moderate brain metastatic prostate cancer DU145 cells (8.1-, 1.8-, and 5.2-fold, respectively). The expression levels of VEGFR-3 were markedly higher in PC-3 cells than in PrEC, LNCaP, and DU145 cells, suggesting that VEGFR-3 is a critical factor influencing the androgen-independent and/or highly metastatic properties of prostate cancer. The expression of VEGFR-3 was previously shown to be involved in embryonic angiogenesis, lymphangiogenesis (Roskoski, 2007; Simons et al., 2016), neural stem cell activation (Han et al., 2015), and cancer progression (Su et al., 2007; Hsu et al., 2019). To date, the expression of VEGFR-3 has been detected in prostate cancer cells (Tsurusaki et al., 1999; Li et al., 2004; Zeng et al., 2004; Jennbacken et al., 2005; Yang et al., 2006; Yang et al., 2014), however, the pathological role of VEGFR-3 signaling remains unclear. Furthermore, the effects of VEGFR-3 kinase inhibitors on the development of prostate cancer have not yet been examined. Therefore, we focused on the up-regulation and activity of VEGFR-3 in human androgen-independent/highly metastatic prostate cancer PC-3 cells in the present study.

In addition to the up-regulation of VEGFR-3 in PC-3 cells, its phosphorylation was strongly facilitated by the stimulation with VEGF-C, a specific ligand for VEGFR-3 (and VEGFR-2) (Ferrara and Adamis, 2016; Hsu et al., 2019), indicating that VEGFR-3 is functional and can mediate its signaling into the cells. This phosphorylation was observed even without the VEGF-C stimulation (Figure 3A), suggesting that the VEGFR-3 signal was constitutively activated in PC-3 cells. VEGF-C binds to VEGFR-3 (and also VEGFR-2) and facilitates its dimerization and phosphorylation. VEGFR-3 signals mainly activate the Akt cascade rather than MAPK pathways (Figure 4), as reported previously (Ferrara and Adamis, 2016; Hsu et al., 2019). This is also supported by the results that 100 nM GSK690693 (an Akt inhibitor, IC_50_ = 2–13 nM) (Rhodes et al., 2008) inhibited the growth of PC-3 cells. Similar to the phosphorylation of VEGFR-3, the phosphorylation of Akt was constitutively activated in PC-3 cells (Figure 6A). The inhibition of the tyrosine kinase of VEGFR-3 by 1–3 μM MAZ51 clearly attenuated the expression and phosphorylation of VEGFR-3 as well as the phosphorylation of Akt. Further studies are required to elucidate the complete molecular mechanism underlying the downregulation of VEGFR-3 expression in response to MAZ51 in PC-3 cells. Although MAZ51 is a tyrosine kinase inhibitor of VEGFR-2 and VEGFR-3, it preferentially inhibits the phosphorylation of VEGFR-3 (lower concentrations at ∼5 μM) over that of VEGFR-2 (higher concentrations at ∼50 μM) (Kirkin et al., 2001). Therefore, lower concentrations of MAZ51 (∼3 μM) were used to inhibit VEGFR-3 in the present study. The pretreatment with 3 μM MAZ51 failed to attenuate the VEGF-C-induced phosphorylation of VEGFR-2 and the expression of VEGFR-2 in PC-3 cells.

VEGF-C was initially cloned from PC-3 cells as a ligand for VEGFR-2 and VEGFR-3 (Joukov et al., 1996). VEGF-C contributes to lymphangiogenesis during embryogenesis, the maintenance of a differentiated lymphatic endothelium in adults (Roskoski, 2007), and osteoclastic bone resorption (Zhang et al., 2008). Moreover, VEGF-C is overexpressed in several cancer types (Su et al., 2007) including prostatic carcinoma (Tsurusaki et al., 1999; Zeng et al., 2004; Jennbacken et al., 2005; Yang et al., 2006; Yang et al., 2014). Plasma levels of VEGF have been correlated with the PSA levels and Gleason scores of patients with prostate cancer (Duque et al., 2006). The VEGF-C ELISA assay revealed that PC-3 cells themselves secreted VEGF-C and potentially activated VEGF-C/VEGFR-3 signaling through an autocrine mechanism, leading to autonomous tumor development in prostate cancer. A similar autocrine mechanism mediated by VEGF-C and VEGFR-3 has been proposed for oral squamous (Matsuura et al., 2009; Shigetomi et al., 2018), ovarian (Decio et al., 2014; Lim et al., 2014), and mammary (Varney and Singh, 2015) carcinomas. It has been reported that the expression of VEGF-C and VEGFR-3 is upregulated in human prostate cancer, which is associated with lymph node metastasis (Tsurusaki et al., 1999; Jennbacken et al., 2005).

The growth and proliferation of PC-3 cells was more rapid than those of PrEC cells. Accelerated cell proliferation was blocked by MAZ51 in a concentration-dependent manner with an IC_50_ value of 2.7 μM. Since this IC_50_ value was consistent with that of MAZ51 for VEGFR-3 (∼5 μM) (Kirkin et al., 2001) and the inhibitory effect of 300 nM VEGFR2 kinase Inhibitor I (IC_50_ = 70 nM) (Sun et al., 1998) on the growth of PC-3 cells was much smaller than that of MAZ51 (compare Figure 7B, c and Figure 7D), the blockade of cell proliferation by MAZ51 was potentially mediated by the inhibition of VEGFR-3. In addition, the proliferation of PC-3 cells reduced by VEGFR-3 siRNA. However, higher concentrations of MAZ51 (>10 μM) slightly affected cell growth even in PrEC cells, suggesting that MAZ51 exerts non-specific effects at concentrations higher than 10 μM. In addition to its antiproliferative effects, MAZ51 and VEGFR-3 siRNA markedly reduced the migration of PC-3 cells stimulated by VEGF-C. The number of migrating cells after the co-treatment with VEGF-C and MAZ51 or VEGFR-3 siRNA was smaller than that of the control. This result (Figure 8) demonstrated that VEGFR-3 was activated, even under the resting state, in PC-3 cells, which may be consistent with our hypothesis of the autocrine effects of VEGF-C and VEGFR-3 in PC-3 cells (Figure 5).

The most important result of the present study showed that the development of tumors transplanted with PC-3 cells was clearly attenuated by the treatment with MAZ51 in the xenograft mouse model. Similar pharmacological effects have been reported for MAZ51 in rat mammary carcinoma MT-450 cells (Kirkin et al., 2004), mouse mammary adenocarcinoma CI66 cells (Varney and Singh, 2015), and mouse melanoma B16-F10 cells (Lee et al., 2016). In addition to these tumors, MAZ51 blocked the tumor growth of androgen-independent/highly metastatic prostate cancer in *ex vivo* and *in vitro* models. The inhibition of prostate cancer cell proliferation and migration is important in drug therapy for prostate cancer. Since tumor growth in the early stages of prostate cancer is dependent on androgens, androgen deprivation therapy successfully induces tumor regression. However, castration-resistant prostate cancer develops following long-term treatment and the advanced metastatic stage of prostate cancer is independent of androgens, which presents clinically difficult challenges for treatment (Hughes et al., 2005; Flourakis and Prevarskaya, 2009; Sathianathen et al., 2018). Therefore, the screening of molecular targets for the treatment of androgen-independent prostate cancer, e.g., VEGFR-3 in the present study, provides important information for elucidating the pathogenesis of prostate cancer and developing novel drugs for its treatment.

In conclusion, the present results demonstrated that VEGFR-3 was functionally expressed in human androgen-independent/highly metastatic prostate cancer PC-3 cells, contributed to cell proliferation and migration, and promoted tumor growth. The VEGFR-3 kinase inhibitor or VEGFR-3 knockdown reduced the proliferation and migration of PC-3 cells and blocked the development of prostate cancer. The blockade of VEGFR-3 by MAZ51 has potential as a novel therapeutic approach for prostate cancer.

## Data Availability

The raw data supporting the conclusion of this article will be made available by the authors, without undue reservation.
